# 

**Published:** 1889-09

**Authors:** 


### CONTENTS.

| *MISCELLANEOUS.* | PAGE |
|---|---|
| Gleanings from Discussions at the Recent Meeting of the I. H. A., | 361 |
| I. What is Contagion? | 361 |
| II. Germs, | 367 |
| III. Arnica or Calendula, | 369 |
| IV. Magnesia-Phosphoricum, | 374 |
| V. Sanicula, | 379 |
| VI. Melilotus Alba, | 380 |
| VII. Transverse Presentation, | 381 |
| VIII. Mastitis, | 384 |
| IX. The Care of the Breasts, | 386 |
| X. The Rochester Hahnemannian Hospital, | 391 |
| On the Relative Worth of Symptoms, with Some Remarks on Borax. | |
| Bœnninghausen, | 392 |
| Borax, | 393 |
| Two Clinical Cases. Clarence N. Payne, M. D., | 398 |
| A Cinnabar Case. E. W. Berridge, M. D., | 399 |
| A Case of Poisoning with Oxalic Acid. Translated by S. L., | 400 |
| *BOOK NOTICES AND REVIEWS.* | |
| Psychology as a Natural Science; Applied to Solution of Occult Psychic Phenomena. By C. G. Raue, M. D., | 401 |
| A Modern Superstition in Disease; the Germ Theory Reconsidered. By Lewis Sanders, | 404 |
| Official Health Bulletin No. 7 of Pennsylvania State Board of Health. | 404 |
| Electrical Distribution of Heat, Light, and Power. By Harold P. Brown, | 405 |
| Repertory to Hering's Condensed Materia Medica, | 405 |
| Guernsey's Bœnninghausen, | 406 |
| *NOTES AND NOTICES.* | |
| Removals—Errata—The New Board of Censors of I. H. A.—The Homœopathic Medical Society of Pennsylvania—The Indiana Institute of Homœopathy—To Builders—I. H. A. Notice—What Are the Remedies—The October and November Numbers, | 406 |

